# Molecular characterization of the effects of essential oils on mRNA gene expression related to proinflammatory and antiviral pathways, virus load, and antibody production in chickens vaccinated against Newcastle disease virus, infectious bursal disease virus, and infectious bronchitis virus

**DOI:** 10.1016/j.psj.2025.106178

**Published:** 2025-12-02

**Authors:** Jaime A. Angel-Isaza, María F. Naranjo-Ortiz, Angi L. Montoya, Diana S. Vargas-Bermúdez, Blanca C. Martínez, Jairo Jaime, Juan C. Rodríguez‑Lecompte

**Affiliations:** a Unidad de Innovación y Desarrollo de Promitec Santander, Bucaramanga (Santander), Colombia; b Universidad Nacional de Colombia, Sede Bogotá. Facultad de Medicina Veterinaria y de Zootecnia. Departamento de Salud Animal. Centro de Investigación en Infectología e Inmunología Veterinaria – CI3V. Carrera 30 No. 45-03. Bogotá D.C. Colombia, Colombia; c Department of Pathology and Microbiology, Atlantic Veterinary College, University of Prince Edward Island, Charlottetown, PE, C1A 4P3, Canada

**Keywords:** Essential oils, Innate immunity, Immunomodulation, Poultry, Cineole

## Abstract

This study evaluated the effects of essential oils (EOs) on pro-inflammatory and antiviral mRNA expression, viral load, and serum antibody levels in broiler chickens, with and without vaccination. Four experiments were conducted using live attenuated vaccines for Newcastle disease virus (NDV), infectious bursal disease virus (IBDV), and infectious bronchitis virus (IBV): EXP1 (unvaccinated control), EXP2 (NDV), EXP3 (IBDV), and EXP4 (IBV). Each experiment included three treatments: T1 (no EO), T2 (a blend of *Lippia origanoides, Rosmarinus officinalis*, and *Lippia alba*), and T3 (*Lippia alba* alone). Birds were vaccinated at 14 days post-hatch, and tracheal and bursal samples were collected on days 21 and 28 to evaluate mRNA expression of NF-κB, IL-1β, TLR3, TLR7, MDA5, IFN-β, OAS, and PKR, as well as viral load and antibody titers. In the trachea, both T2 and T3 modulated antiviral gene expression in EXP1 and EXP2, with significant up-regulation of TLR3, IFN-β, and OAS on day 21, as well as additional effects on day 28. In EXP4, OAS was up-regulated by both treatments on day 21, while TLR3, MDA5, and PKR were enhanced on day 28. In the bursa, IFN-β was up-regulated in EXP1 on day 21 and broader activation occurred by day 28. In EXP3, TLR7 increased on day 21, and TLR3 and MDA5 (T2) on day 28. Viral load decreased with T3 in NDV (EXP2) at both time points and in IBDV (EXP3) on day 21, with no significant change for IBV (EXP4). Antibody titers were unchanged on day 21 but increased on day 28 for NDV and IBDV (T2 and T3) and for IBV (T3). These findings suggest that EO supplementation via spray application may modulate antiviral pathways, reduce viral replication, and enhance humoral responses, supporting its potential as a natural immunomodulator to improve antiviral protection in poultry.

## Introduction

Newcastle disease virus (**NDV**), infectious bursal disease virus (**IBDV**), and infectious bronchitis virus (**IBV**) are significant pathogens affecting the global poultry industry (Alexander, 2001; El-Shall et al., 2020; Li et al., 2020). NDV, a negative-sense, single-stranded RNA virus, causes Newcastle disease, characterized by respiratory distress, neurological signs, and high mortality rates in chickens, resulting in severe economic losses due to decreased production and trade restrictions (Alexander, 2001). IBDV, a double-stranded, segmented RNA virus, causes infectious bursal disease (Gumboro disease), primarily targeting the bursa of Fabricius in young chickens, resulting in immunosuppression and increased susceptibility to secondary infections (Rodríguez‑Lecompte et al., 2005; Rautenschlein and Alkie, 2016; Zhang et al., 2022). The emergence of very virulent strains has further exacerbated its impact globally. IBV, a positive-sense, single-stranded RNA virus, causes infectious bronchitis, an acute and highly contagious disease affecting the respiratory tract, kidneys, and reproductive systems (Cavanagh, 2005). It reduces egg production and quality, with multiple strains and serotypes complicating control measures (Jackwood, 2012; Bande et al., 2017; Hartenian et al., 2020). Effective management of these diseases relies on stringent biosecurity practices and region-specific vaccination programs.

The innate antiviral response plays a central role in detecting and controlling RNA viruses in poultry. Viral RNA is sensed in the cytoplasm by the RIG-I-like receptors RIG-I and MDA5, and in endosomes by TLR3 (dsRNA) and TLR7/8 (ssRNA) (Rehwinkel and Gack, 2020; Kawai and Akira, 2010). These receptors signal through MAVS or TRIF/MyD88 to activate TBK1 and IKKε, leading to the phosphorylation of IRF3 and IRF7, the production of type I IFN (IFN-α/β), and the activation of NF-κB–driven inflammatory cytokines (Wu and Hur, 2015; Kumar et al., 2011). Secreted IFNs then engage IFNAR to initiate JAK-STAT signaling and induce ISGs such as PKR, OAS, and MX1, which restrict viral replication (Ivashkiv and Donlin, 2014). These pathways are critical in host defence against NDV, IBDV, and IBV: NDV triggers RLR and TLR3/7 signaling (Xu et al., 2021), IBDV inhibits IFN-β through VP3 but is countered by TLR3/MDA5 responses (Ye et al., 2014), and IBV disrupts IFN signaling yet remains controlled through JAK-STAT–mediated ISG activation (Kint et al., 2015). Together, these mechanisms define the core framework of early antiviral immunity in poultry.

Essential oils (EOs) have garnered growing interest due to their antiviral effects against poultry viruses (Brenes and Roura, 2010). EOs from Syzygium aromaticum, Origanum vulgare, Cymbopogon martinii, and Cymbopogon citratus can completely inhibit avian coronavirus replication at a 1 % dilution (Okino et al., 2024). Likewise, a eucalyptus–peppermint blend exhibits virucidal activity against avian influenza virus (AIV) and NDV, achieving complete inactivation at 2.78 % and 13.9 %, respectively, after 30 min of exposure (Barbour et al., 2010). Despite these promising effects, most EO research in poultry has focused on performance, antibody responses, or reductions in viral load, leaving a critical gap in understanding how EOs influence innate antiviral immunity, particularly PRR signaling, type I IFN induction, and ISG activation.

Previous studies in our laboratory were conducted as a proof of concept for the use of EO as immunomodulators and to determine their concentrations in chicken macrophages. These results showed that the use of Lippia origanoides and Rosmarinus officinalis, either singly or in a blend combination ranging from 10 to 1000 ppm, challenged vaccine (MOI = 1) of IBDV and innate immunity genes (IL-1β, IL-6, IL-12, IL-10, IRF-7, IFN-β, OAS, and PKR) (Uribe-Diaz et al., 2019; Rodriguez-Lecompte et al., 2020). These findings suggest that incorporating specific EOs into poultry health management strategies could provide a natural means of mitigating viral infections. However, further research is needed to optimize their efficacy and practical applications. In this context, this study aimed to assess the potential of spray-administered EOs to modulate innate immune signaling pathways, thereby influencing gene expression profiles, viral replication, and humoral immune responses in both unvaccinated and vaccinated chickens against NDV, IBDV, and IBV.

## Materials and methods

### Essential oils and chemical characterization

Essential oils (EOs) were extracted from *Lippia origanoides, Lippia alba*, and *Rosmarinus officinalis* using microwave-assisted hydrodistillation, following the method described by Moradi et al. (2018). Leaves and stems were air-dried under dark conditions, ground, and homogenized before extraction. For each plant, 100 g of dried material was distilled with 500 mL of water for 3.5 h in a Clevenger-type apparatus operating at atmospheric pressure and temperatures above 180 °C. Following condensation, the EO phase was separated based on differences in volatility and immiscibility. The extracted EOs were incorporated into a proprietary oil-in-water (o/w) emulsion (Promitec et al., 2020). The formulation was stabilized using emulsifiers to maintain the hydrophilic–lipophilic balance, ensuring droplet stability and long-term homogeneity.

Chemical characterization of the individual EOs and the final emulsion was performed by gas chromatography coupled to mass spectrometry (**GC–MS**) at the National Center for the Agroindustrialization of Aromatic and Medicinal Tropical Plant Species (CENIVAM), Industrial University of Santander (Bucaramanga, Colombia). Analyses were conducted using electron ionization (EI, 70 eV) and both polar and non-polar capillary columns, as described by Stashenko et al. (2012). Component identification was based on linear retention indices and mass spectral data, and the relative abundances of major compounds (greater than 1 %) were determined. The complete formulation was primarily composed of monoterpenes (57 %), monoterpenoids (29 %), and sesquiterpenoids (14 %). Table 1 summarizes the principal volatile constituents of the EO blend and the *Lippia alba* EO emulsion used in this study.

**Table 1:** Relative abundance (%) of major compounds (>1 %) identified by GC–MS in the essential oil emulsions used in this study.

**Plant**	**Main volatile secondary metabolites**
T2 - EO blend	1,8-cineole (7.3), camphor (3.9), geranial (3.5), β-pinene (2.1), thymol (2.3), carvacrol (1.3), α-pinene (2.5), *p*-cymene (2.5), trans-β-caryophyllene (0.6) and geraniol (0.4)
T3 - *Lippia alba*	neral + geranial (26.1), geraniol (2.9) and trans-β-caryophyllene (2.7)

### General study design

Six hundred one-day-old male Ross broiler chickens were obtained from a local commercial hatchery and housed in the experimental farm (Bucaramanga, Colombia), which were divided into four separate experiments (**EXP**) using different viral vaccines virus, EXP1 (control no virus), EXP2 (Newcastle disease virus), EXP3 (Infectious bursal disease virus) and EXP4 (Infectious bronchitis virus). Each experiment included 150 animals, further randomly distributed into three different treatment groups (T1 control, T2 EO blend, *T3 Lippia alba*). Each treatment consisted of 50 chickens housed in 5 pens with 10 animals per pen, ensuring five biological replications per treatment. Sample size was determined in RStudio using the ‘pwr’ package for one-way ANOVA to achieve 80 % statistical power at α = 0.05, specifying Cohen’s f (*f* = 0.43) as the effect size, estimated from previous studies conducted by our research group (Lakens, 2022). The final allocation was consistent with Mead’s resource equation (Mead, 1988) and explicitly aligned with the 3Rs principle of animal bioethics (Lauwereyns et al., 2024), aiming to minimize the use of animals.

Water and feed were provided *ad libitum*. This experimental design enabled a controlled evaluation of the different treatments while maintaining consistency and statistical reliability throughout the study. This study was conducted in accordance with the guidelines of the University of Prince Edward Island Animal Care Committee, and the birds were cared for in accordance with the recommendations established by the Canadian Council on Animal Care (CCAC, 2010).

**Fig. 1:**
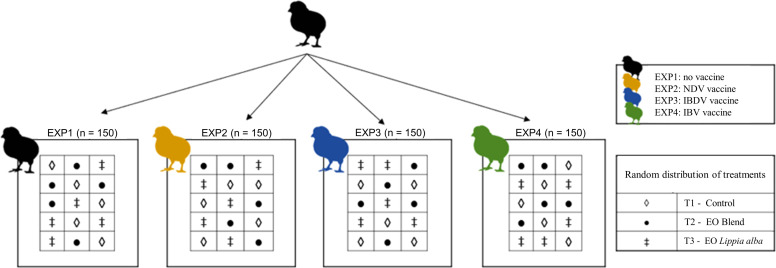
Schematic representation of the experimental design of the study. Six hundred one-day-old broiler chickens Ross AP were distributed in four experiments (EXP) (150 chickens for each Experiment) [EXP1: no vaccine; EXP2: NDV vaccine virus (NOBILIS ND clone 30); EXP3: IBDV vaccine virus (Hipragumboro strain Winterfield clone CH/80), and EXP4: IBV vaccine virus (Hipraviar H120)]. The 150 chickens were randomly distributed in the 15 pens (ten chickens per pen), considering each pen an experimental unit. In each EXP, the following treatments were applied: T1 (Control): distilled water as a spray; T2 (EO blend): EO mixture (1,500 ppm in spray); T3 (EO *Lippia alba*): *Lippia alba* essential oil (1,500 ppm in spray).

Four independent experiments (EXP1–EXP4) were conducted (Fig. 1), each corresponding to a different commercial live viral vaccine administered by a single ocular dose at 14 days of age, except for the unvaccinated control (EXP1). The experimental design was as follows: EXP1, unvaccinated control; EXP2, Newcastle disease virus (NDV) vaccine (Nobilis ND LaSota Clone 30, 6.0 log₁₀ EID₅₀ dose); EXP3, infectious bursal disease virus (IBDV) vaccine (Hipragumboro, Winterfield CH/80 clone, 3.5 log₁₀ TCID₅₀ dose); and EXP4, infectious bronchitis virus (IBV) vaccine (Hipraviar Massachusetts H120, 6.5 log₁₀ EID₅₀ dose). Within each experiment, three treatment groups were evaluated: T1 (control): distilled water spray; T2 (EO blend): a formulation containing *Lippia origanoides, Rosmarinus officinalis*, and *Lippia alba* at 1,500 ppm; and T3 (single EO): *Lippia alba* essential oil at 1,500 ppm. Treatments were administered once daily from 13 to 16 days of age using a 20-L backpack sprayer (Royal Condor, model CO-048) fitted with a brass nozzle (reference RC-350A101X). The sprayer operated at 50 psi ± 10 %, delivering approximately 600 mL/min with droplet sizes ranging from 250 to 350 µm, as specified by the manufacturer. Each spray application lasted approximately 5 seconds per cage, ensuring the uniform dispersion of fine droplets throughout the rearing area. All pens were treated under identical environmental and ventilation conditions to guarantee consistent exposure among groups, and physical barriers were used to prevent cross-contamination during application. EO formulations were freshly prepared according to the manufacturer’s recommendations and diluted in water to a final concentration of 1,500 ppm immediately before use with the spray system.

### Samples

In each of the four experiments, five chickens per treatment (one per pen) were euthanized at 21 and 28 days of age for sample collection. The euthanasia process followed the guidelines outlined in the Canadian Council on Animal Care (CCAC, 2010) for the use of farm animals in research. Subsequently, a necropsy was performed, and tissue samples were collected from the trachea and the Bursa of Fabricius. These tissues were individually placed in Eppendorf tubes containing TRIzol Reagent (Thermo Fisher Scientific) at a ratio of 0.5 g of tissue to 750 µL of TRIzol. The tubes were then refrigerated at 4°C and stored at −80°C until processing. In each experiment, three chickens per pen (experimental unit; total of 15 chickens per treatment) were selected for antibody (Ab) detection at 21 (7 days post-vaccination or **dpv**) and 28 (14 dpv) days of age, and 1 mL blood samples were collected via brachial vein puncture. The blood was stored in refrigerated Eppendorf tubes, centrifuged at 3,000 x g in a microcentrifuge, and the serum was stored at −20 °C until further processing.

### RNA extraction from tissue samples

For RNA extraction, tissue samples were thawed at room temperature. Then, 0.5 g was weighed and homogenized in 750 µL of TRIzol reagent using a Fisher brand 150 Handheld Homogenizer Motor (Pittsburgh, PA, USA). The homogenate was collected in an Eppendorf tube and incubated for 5 min at room temperature. Subsequently, 200 µL of chloroform was added, the mixture was incubated for 3 min, and then centrifuged at 12,000 × *g* for 15 min at 4 °C. The aqueous phase was transferred to a new tube, and 500 µL of 100 % isopropanol was added. The mixture was then incubated at room temperature for 10 min and subsequently centrifuged at 12,000 x g for 10 min at 4 °C. The pellet was homogenized, treated with 1 mL of 75 % ethanol, and centrifuged at 7,500 xg for 15 min at 4 °C. The supernatant was discarded, and the pellet was allowed to dry for 7 min at room temperature. The pellet was homogenized with 100 µL of RNase-free molecular grade H_2_O for RNA solubilization and incubated at 60 °C for 15 min. Once the RNA was recovered, it was quantified by measuring the OD260 on a Nano200 spectrophotometer (Thermo Scientific, Wilmington, Delaware, USA) and stored at −70 °C for subsequent use.

### cDNA synthesis and relative quantification of genes associated with innate immunity

Subsequently, cDNA synthesis was carried out for all samples using a High-Capacity cDNA Reverse Transcription Kit (Applied Biosystems, Thermo Fisher Scientific, Vilnius, Lithuania) on a Bio-Rad DNA thermocycler (Hercules, CA, USA). The protocol used was 1x buffer, 4 Mmol of deoxyribonucleotide triphosphates, 1x random primers, 1x RNase inhibitor, and 2 U of multiscribe reverse transcriptase; the volumes of the reagents were those recommended by the manufacturer. The RT conditions were one cycle at 25 °C for 10 min, one at 37 °C for 120 min, and one at 85 °C for 5 min. The cDNA was stored at −20 °C for subsequent use.

### Cloning and amplification of plasmids

Standard curves for real-time PCR and internal controls were generated by cloning specific gene sequences into plasmids. These constructs were prepared as described previously (Jaime et al., 2020) at the Immunology Laboratory of the Atlantic Veterinary College, University of Prince Edward Island, PEI, Canada. Dr. Juan Carlos Rodríguez-Lecompte kindly donated them. Briefly, sequences of immunity-associated genes—such as the endosomal sensors TLR3 and TLR7; the cytoplasmic sensor MDA5; the transcription factor NF-κB; the cytokines IL-1β and IFN-β; and the antiviral proteins OAS and PKR—along with viral genes (IBV-ORF1, IBDV-VP2, and NDV-M) were amplified using specific primers (Table 2) via conventional PCR (RT-PCR was performed for IBDV-VP2 and NDV-M). Each PCR reaction contained 12.5 μL of 1× Promega Master Mix, 1 μL of 1 μmol primer, and 2 μL of DNA. Thermal cycling conditions included an initial denaturation at 94 °C for 2 min, followed by 35 cycles of 94 °C for 30 s, 55 °C for 45 s, 72 °C for 45 s, and a final extension at 72 °C for 5 min. Amplicon sizes were confirmed by electrophoresis on a 1 % agarose gel. Cloning was performed using the Invitrogen TOPO TA Cloning Kit (ThermoFisher Scientific, Burlington, ON, Canada) following the manufacturer’s instructions.

**Table 2:** The sequence of the primers used to detect the expression of genes associated with innate immunity, the viruses studied, and a constitutive gene.

Gene name	Primer sequence	Annealing temperature (°C)	Amplicon size (bp)	Accession number or reference
β-actin	F: 5′-CAACACAGTGCTGTCTGGTGGTA-3′	61	205	X00182
	R: 5′-ATCGTACTCCTGCTTGCTGATCC-3′			
TLR-3	F: 5′-TGCATAAGAAGGAGCAGGAAG-3′	60	263	NM001011691
	R: 5′-CTGGCCAGTTCAAGATGCAG-3′			
TLR-7	F: 5′-TTCTGGCCACAGATGTGACC-3′	60	218	MN313017
	R: 5′-CCTTCAACTTGGCAGTGCAG-3′			
NF-κβ	F: 5′-CATTGCCAGCATGGCTACTAT-3′	59	101	D13721
	R: 5′-TTCCAGTTCCCGTTTCTTCAC-3′			
IL-1β	F: 5′-AACCCGACCAGGTCAACA-3′	61	101	AJ245728.1
	R: 5′-CGGTACATACGAGATGGAAAC-3′			
MDA5	F: 5′-GCAAAACCAGCACTGAATGGG-3′	59	178	(Alkie et al., 2015)
	R: 5′-CGTAAATGCTGTTCCACTAACGG-3′			
IFN-β	F: 5′-GCCTCCAGCTCCTTCAGAATACG-3′	60	223	(Alkie et al., 2015)
	R: 5′-CTGGATCTGGTTGAGGAGGCTGT-3′			
OAS	F: 5′-AGAACTGCAGAAGAACTTTGTC-3′	58	91	(Alkie et al., 2015)
	R: 5′-GCTTCAACATCTCCTTGTACC-3′			
PKR	F: 5′-GGAGGCGGGAATGGAGAAAA-3′	60	144	(Alkie et al., 2015)
	R: 5′-GAGCACATCCGCAGGTAGAG-3′			
IBDV-VP2	F: 5′-CTGACTACCGGCATCGACA-3′	60	149	AF498631
	R: 5′-CCACTTGCCGACCATGA-3′			
NDV-M	F: 5′-AGTGATGTGCTCGGACCTTC-3′	58	120	MW285790
	R: 5′-CCTGAGGAGAGGCATTTGCTA-3′			
IBV-ORF1	F: 5′-GCTTTTGAGCCTAGCGTT-3′	60	142	OQ189491
	R: 5′-GCCATGTTGTCACTGTCTATTG-3′			

### Standardization of curves for real-time PCR

A standard curve was established for each of the 12 genes by performing 10-fold serial dilutions of the corresponding plasmid. The SsoAdvanced Universal SYBR Green Supermix kit (Hercules, CA, USA) was used for all real-time PCR reactions. Standard curves for each gene were generated using 5 μL of 1× Supermix, 0.2 μL of each primer (1 μmol), and 1 μL of DNA in a total reaction volume of 10 μL. qPCR was performed on a LightCycler 480 instrument (Roche, Burgess Hill, UK). All samples were analyzed by real-time PCR to amplify β-actin, TLR3, TLR7, MDA5, IL-1β, NF-κB, IFN-β, OAS, PKR, NDV, IBDV and IBV, with β-actin serving as the housekeeping gene. Amplification was carried out using the manufacturer’s recommended protocol, with thermal cycling conditions as follows: initial denaturation at 95 °C for 5 min; 35 cycles of denaturation at 95 °C for 1 min, annealing at 58–61 °C for 15 s, and extension at 72 °C for 2 s; followed by a melting curve analysis from 70 to 90 °C to verify specificity. All samples, including positive and negative controls, were run in triplicate.

### Assessment of viral loads for newcastle disease virus (NDV), infectious bursal disease virus (IBDV), and infectious bronchitis virus (IBV)

Real-time RT-PCR assays were conducted using the SsoAdvanced Universal SYBR Green Supermix from BIORAD (Bio-Rad, Hercules, CA, USA) on a LightCycler 480 Real-Time PCR instrument (Roche, Germany) to detect and quantify IBV, IBDV and NDV. Specific primer sets were designed for each virus. For IBDV, the primers IBDV-F (5´-CTGACTACCGGCATCGACA-3′) and IBDV-R (5´-CCACTTGCCGACCATGA-3′) target the VP2 gene, generating a 149-bp amplicon (Li et al., 2005). For IBV, the primers IBV-F (5´-GCTTTTGAGCCTAGCGTT-3′) and IBV-R (5´-GCCATGTTGTCACTGTCTATTG-3′) amplify a 143 bp fragment of the 5′UTR region (Callison et al., 2006). For NDV, the primers NDV-F (5´-AGTGATGTGCTCGGACCTTC-3′) and NDV-R (5´-CCTGAGGAGAGGCATTTGCTA-3′) target the matrix (M) gene, with an expected product size of 121 bp (Wise et al., 2004). The reactions were conducted in a final volume of 20 µL, which included 10 µL of 2x SYBR Green Master Mix (BIORAD), 0.4 µM of each primer, 1 µL of cDNA template, and nuclease-free water. Amplification was carried out under the following thermal cycling conditions: initial denaturation at 95 °C for 10 min, followed by 40 cycles consisting of denaturation at 95 °C for 60 s, annealing at 60 °C for 25 s, and extension at 72 °C for 2 s. After amplification, melting curve analysis was performed by increasing the temperature from 70 °C to 95 °C in increments of 0.5 °C every 5 s. This step was taken to confirm the specificity of the amplicons by observing a single sharp melting peak for each target.

To create positive controls and standardize real-time PCR for each virus, we used attenuated vaccines amplified through conventional PCR. The amplicons were generated using the specified primers and then cloned into the TOPO TA plasmid cloning kit (Invitrogen, Carlsbad, CA, USA). The cloning was transformed into One Shot chemically competent *E. coli*. The presence of the insert and its correct orientation were verified through sequencing at SSiGMol (Servicio de Secuenciación y Análisis Molecular, Instituto de Genética, Universidad Nacional de Colombia, Bogotá). The recombinant plasmids were purified using the Plasmid Maxi Kit (Qiagen, Hilden, Germany) and quantified at 260 nm using a Nano200 spectrophotometer (Thermo Scientific, Wilmington, DE, USA). The plasmids were then diluted in RNase-free H2O to achieve a stock concentration of 108 copies of plasmid DNA per microliter. Standard dilutions were prepared from this stock through serial dilutions in RNase-free H2O, using a logarithmic scale (Log10). From the standard curves, a threshold cycle (**Ct**) < 35 was established as the detection limit, indicating a positive result for each virus. The specificity of each real-time PCR assay was determined using recombinant plasmids constructed for each virus. The limit of detection (LoD) for qPCR, along with the coefficient of determination and efficiency, was assessed using a serial 10-fold dilution curve. The efficiency of the qPCR (E) was evaluated using the formula *E* = 10(−1/slope) − 1. The slope was determined from a linear regression analysis of the crossing points (Ct) against the corresponding log-transformed viral titers. The coefficient of determination (R²) indicates how well the regression line fits the data, summarizing the relationship between dilution and Ct values. Viral quantification data were expressed as log₂ fold change, with Control at 14 days as the calibrator.

### Detection of antibodies

Antibody titers against NDV, IBDV, and IBV were evaluated post-vaccination using hemagglutination inhibition (HI) and ELISA assays. Serum samples were collected at 7 and 14 days post-vaccination (dpv). For NDV, the HI test was conducted in V-bottom microplates following standard procedures (Löndt and Alexander, 2016). Heat-inactivated sera (56 °C for 30 min) were serially twofold diluted and mixed with 10 hemagglutinating units (HAU) of NDV antigen, previously confirmed by titration. After a 30-minute incubation at room temperature, 1 % chicken red blood cells (washed at least three times with PBS) were added. The HI titer was defined as the reciprocal of the highest serum dilution showing complete inhibition of agglutination (compact RBC button) and expressed as GMT log₂ values.

For IBDV and IBV, antibody levels were determined by indirect ELISA using the IDEXX IBD-XR and IBV Ab Test kits (IDEXX Laboratories, Westbrook, ME, USA). Serum samples were diluted 1:500 and processed according to the manufacturer’s instructions. Briefly, diluted samples were incubated for 30 min in antigen-coated wells, washed three times with the kit wash buffer, and then reacted with horseradish peroxidase (HRP)–conjugated anti-chicken IgG. Following the addition of TMB substrate, the reaction was stopped with sulfuric acid. And absorbance was measured at 450 nm using a microplate reader. Antibody titers were calculated as sample-to-positive (S/P) ratios and interpreted according to the manufacturer’s cut-off criteria.

### Statistical analysis

Statistical analyses were performed independently for each experiment to evaluate the effects of the three treatments. Results are presented as means ± standard error of the mean (SEM). For gene expression, relative expression levels were normalized to the constitutive gene (β-actin) using the ΔΔCt method (Livak and Schmittgen, 2001) and the ‘PCR’ package in RStudio version 4.3.0 (Ahmed and Kim, 2018). Prior to ANOVA, normality and homoscedasticity of model residuals were verified using the Shapiro–Wilk and Levene’s tests, respectively. One-way ANOVA (*p* < 0.05) was then applied to evaluate differences among treatments for gene expression and antibody titer variables. Tukey’s honestly significant difference (**HSD**) test was used for pairwise comparisons using the agricolae package in R (version 1.3-7.). Where appropriate, P-values were adjusted for multiple testing using the Holm method.

## Results

### Expression of genes associated with the antiviral innate immune response

**Fig. 2:**
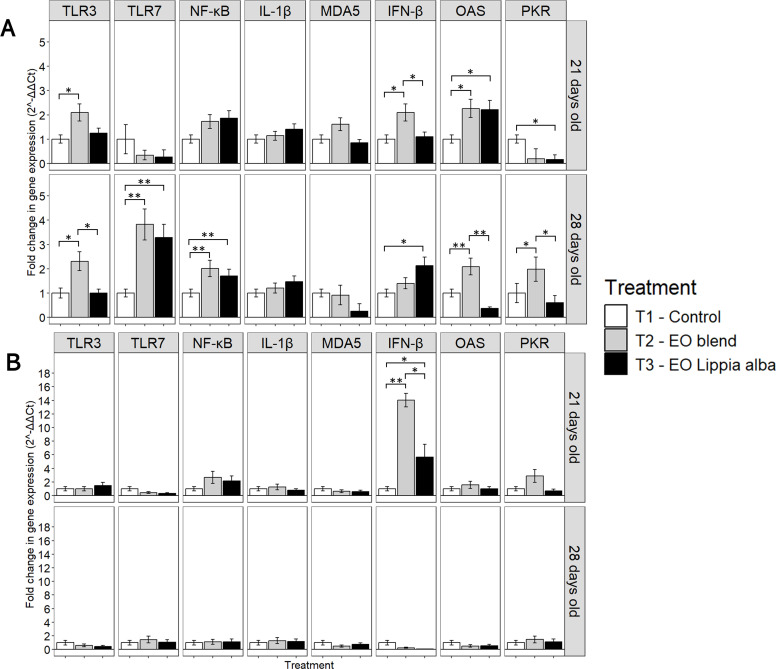
Relative mRNA expression (2⁻ΔΔCt) of innate immune-related genes in chickens from Experiment 1 (EXP1, non-vaccinated group). (A) Trachea tissue; (B) Bursa of Fabricius. Samples were collected at 21 (7 dpv) and 28 days of age (14 dpv). Bars connected by * or ** denote significant differences (*p* < 0.05 and *p* < 0.001, respectively) among treatments within each gene, based on one-way ANOVA (α = 0.05) followed by Tukey’s HSD multiple comparison test. TLR3, Toll-like receptor 3; TLR7, Toll-like receptor 7; MDA5, melanoma differentiation-associated gene 5; NF-κB, nuclear factor κB; IL-1β, interleukin 1β; IFN-β, interferon β; OAS, 2′−5′-oligoadenylate synthetase; PKR, protein kinase R; EO, essential oil.

EXP 1: Without Vaccine. The immune response, as evaluated in tracheal tissue, showed that at day 21, the EO blend (T2) significantly increased the expression of immune-related genes, including TLR3 (*p* = 0.031), IFN-β (*p* = 0.009), and OAS (*p* = 0.029). *Lippia alba* (T3) also increased OAS (*p* = 0.016) expression. By day 28, T2 continued to show elevated OAS levels (*p* = 0.018), along with a significant increase in TLR7 (*p* = 0.028) and NF-κB (*p* = 0.045). However, in T2, IFN-β levels slightly declined. In contrast, T3 significantly increased TLR7 (*p* = 0.044), NF-κB (*p* = 0.046) and IFN-β (*p* < 0.001). By day 28, TLR7 expression had increased 3.8-fold in T2 and 3.2-fold in T3. NF-κB levels rose in both groups. However, while OAS remained elevated in T2, it declined in T3 by day 28 (Fig. 2A).

Regarding the Bursa of Fabricius response, on day 21, both T2 and T3 showed a significant increase in IFN-β expression (*p* < 0.001 and *p* = 0.014, respectively). However, by day 28, no significant differences in gene expression were observed. Over time, IFN-β levels, which had been strongly up-regulated at day 21 (T2: 14-fold, T3: 5.6-fold), returned to baseline by day 28 (Fig. 2B).

**Fig. 3:**
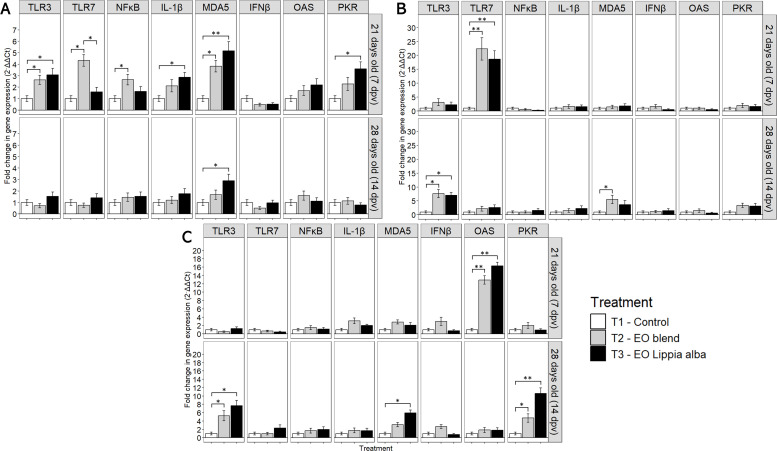
Relative mRNA expression (2⁻ΔΔCt) of innate immune-related genes in chickens from EX 2–4 vaccinated with different live viral vaccines. (A) EXP2 (NDV, trachea); (B) EXP3 (IBDV, bursa of Fabricius); (C) EXP4 (IBV, trachea). Samples were collected at 21 (7 dpv) and 28 days of age (14 dpv). Bars connected by * or ** denote significant differences (*p* < 0.05 and *p* < 0.001, respectively) among treatments within each gene, based on one-way ANOVA (α = 0.05) followed by Tukey’s HSD multiple comparison test. TLR3, Toll-like receptor 3; TLR7, Toll-like receptor 7; MDA5, melanoma differentiation-associated gene 5; NF-κB, nuclear factor κB; IL-1β, interleukin 1β; IFN-β, interferon β; OAS, 2′−5′-oligoadenylate synthetase; PKR, protein kinase R; EO, essential oil.

EXP 2: NDV Vaccine. The immune response, as evaluated in tracheal tissue, showed that at 7 dpv, T2 significantly increased the expression of TLR3 (*p* = 0.046), TLR7 (*p* = 0.003), and MDA5 (*p* = 0.009). T3 also up-regulated TLR3 (*p* = 0.028) and MDA5 (*p* = 0.002) and significantly increased IL-1β (*p* = 0.045) and PKR (*p* = 0.020). By day 28 (14 dpv), no significant changes (*p* > 0.05) in gene expression were observed in the trachea (Fig. 3A).

EXP 3: IBDV Vaccine. Analysis of the immune response in the Bursa of Fabricius revealed that at day 21 (7 dpv), both T2 and T3 significantly up-regulated TLR7 (*p* < 0.001 and *p* < 0.001) expression. By day 28 (14 dpv), TLR3 expression increased, replacing TLR7 activation. Additionally, MDA5 was significantly elevated (*p* = 0.024) in T2. Over time, TLR7 activation at day 21 was replaced by TLR3 and MDA5 up-regulation by day 28 (Fig. 3B).

EXP 4: IBV Vaccine. Immune gene expression analysis in tracheal tissue showed that at day 21 (7 dpv), T2 and T3 significantly increased OAS (*p* = 0.012 and *p* = 0.001) expression. By day 28 (14 dpv), TLR3 expression was elevated in both T2 (*p* = 0.048) and T3 (*p* = 0.046), while T3 significantly increased MDA5 (*p* = 0.008) and PKR (*p* = 0.004) levels. Over time, viral recognition genes (TLR3, MDA5) and antiviral proteins (OAS, PKR) increased, with T2 exhibiting a stronger overall response (Fig. 3C).

### Viral load analysis

**Fig. 4:**
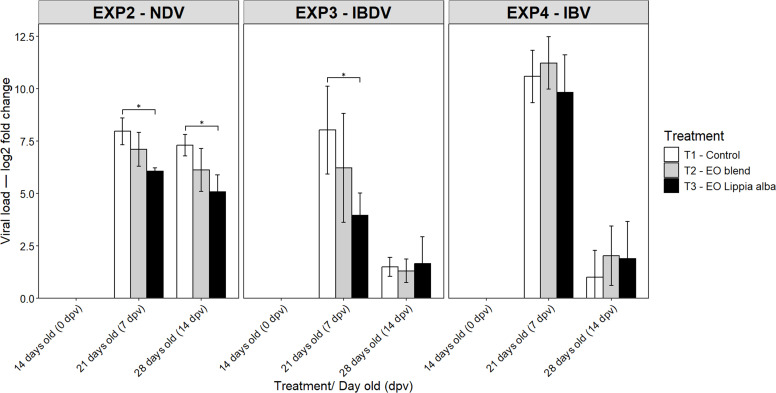
Viral loads (log₂ fold change) in trachea (NDV and IBV) and bursa of Fabricius (IBDV) from chickens at 14 (0 dpv), 21 (7 dpv), and 28 d of age (14 dpv). Viral quantification was performed using the 2⁻ΔΔCt method, and data are expressed as log₂ fold change relative to the control group at 14 d of age (calibrator). Bars connected by * or ** indicate significant differences (*P* < 0.05 and *P* < 0.001, respectively) among treatments within each virus, based on one-way ANOVA (α = 0.05) followed by Tukey’s HSD multiple comparison test. NDV, Newcastle disease virus; IBDV, infectious bursal disease virus; IBV, infectious bronchitis virus; EO, essential oil.

Analysis of relative viral loads (log₂ fold change) revealed a transient antiviral effect of *Lippia alba* essential oil against NDV and IBDV (Fig. 4). In NDV-vaccinated chickens, tracheal viral load was significantly lower in the *Lippia alba* group compared with controls at 7 dpv (*p* = 0.009) and 14 dpv (*p* = 0.045). In contrast, the EO blend did not differ significantly from controls at either time point (*p* = 0.102 and *p* = 0.318 for 7 and 14 dpv, respectively). Overall, NDV viral loads increased at 7 dpv and remained elevated through 14 dpv across treatments. For IBDV, analyzed in the bursa of Fabricius, a significant reduction in viral load was observed in chickens treated with *Lippia alba* essential oil (*p* = 0.028) compared with the control at 7 dpv, whereas no significant differences were detected for the EO blend compared with the control (*p* = 0.102 and *p* = 0.318 for 7 and 14 dpv, respectively). Overall, IBDV viral loads tended to decrease by 14 dpv in all groups, indicating a lower level of viral replication toward the end of the evaluation period. In the case of IBV, tracheal viral load did not differ significantly among treatments at any sampling point; however, all groups showed a general decline in viral load at 14 dpv compared with 7 dpv.

### Antibody response

**Fig. 5:**
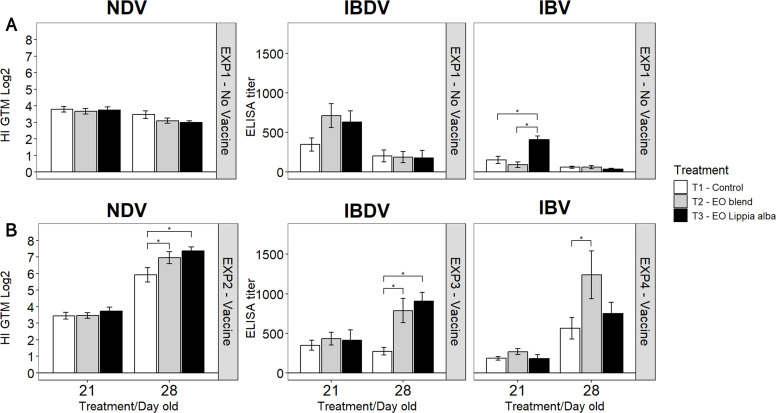
Antibody titers against NDV, IBDV, and IBV in chickens from the four experimental groups. (A) EXP1 (non-vaccinated birds), (B) EXP2–4, birds vaccinated with NDV, IBDV, or IBV, respectively. Samples were collected at 21 (7 dpv) and 28 days of age (14 dpv). NDV antibodies were measured by hemagglutination inhibition (HI) and expressed as log₂ HI titers. IBDV and IBV antibodies were quantified by ELISA and expressed as sample-to-positive (S/P) ratios. Bars connected by * or ** indicate significant differences (*p* < 0.05 and *p* < 0.001, respectively) among treatments within each virus, based on one-way ANOVA (α = 0.05) followed by Tukey’s HSD multiple comparison test. NDV, Newcastle disease virus; IBDV, infectious bursal disease virus; IBV, infectious bronchitis virus; EO, essential oil.

In the non-vaccinated chickens (EXP1), antibody levels against all viruses tended to decrease between 21 and 28 days of age. However, in the case of IBV, chickens treated with *Lippia alba* essential oil showed significantly higher antibody titers (*p* = 0.032) than the control group at 21 days of age, indicating a transient reduced antibody decay (Fig. 5a)

In NDV-vaccinated chickens (EXP2), antibody titers increased in all groups by 14 dpv, reflecting the expected rise in the post-vaccination humoral response. Nevertheless, birds treated with *Lippia alba* and EO blend essential oils exhibited significantly higher titers (*p* = 0.022 and *p* = 0.029, respectively) compared with the control group at 14 dpv. For IBDV (EXP3), antibody titers in the control group did not increase between 7 and 14 dpv, unlike the chickens treated with *Lippia alba* and EO blend essential oils, which showed significantly higher antibody levels at 14 dpv than the control group (*p* = 0.001 and *p* = 0.006, respectively). Finally, for IBV (EXP4), no differences were observed at 7 dpv; however, at 14 dpv, chickens treated with *Lippia alba* essential oil showed higher antibody production than the control group (*p* = 0.044), consistent with the general increase in titers observed during this post-vaccination stage (Fig. 5b).

## Discussion

Vaccination against Newcastle disease virus (NDV), infectious bursal disease virus (IBDV), and infectious bronchitis virus (IBV) remains critical in poultry production, as these RNA and dsRNA viruses cause high mortality, immunosuppression, and production losses by impairing innate and adaptive immunity (Zhang et al., 2023; Wegner et al., 2025). Enhancing both local and systemic immune responses through immunomodulators during infection and vaccination can improve antigen recognition, antibody production, and long-term protection (Bodman-Harris et al., 2024; Wlaźlak et al., 2023; Munang’andu, 2024).

EOs have emerged as promising natural immunomodulators in both humans and animals. These complex mixtures of volatile terpenes, phenolics, and oxygenated compounds can modulate host immunity while exerting antimicrobial and antiviral effects (Abd El-Hack et al., 2022; Valdivieso-Ugarte et al., 2019). In poultry, EOs act as functional feed additives that influence pattern recognition receptors (PRRs)—including Toll-like receptors (TLRs)—and regulate NF-κB signaling and cytokine gene expression, thereby shaping pro-inflammatory and regulatory immune responses (Lee et al., 2019; Lillehoj et al., 2018). However, few studies have explored the molecular mechanisms underlying EO-mediated modulation of antiviral pathways in key immune tissues such as the trachea and the bursa of Fabricius.

### EXP1 – unvaccinated chickens

In the present study, spray administration of EOs significantly modulated innate antiviral signaling in the trachea of unvaccinated chickens. Early up-regulation of TLR3, IFN-β, OAS, and PKR was observed at day 7 post-treatment, followed by a broader wave that included TLR7, NF-κB, and sustained induction of OAS and PKR at day 14. The early engagement of TLR3 in the EO blend (T2) group is consistent with the architecture of avian antiviral sensing, in which RIG-I is absent, and chickens depend on coordinated TLR3–MDA5 recognition of viral dsRNA. This reliance enables endosomal TLR3 to rapidly initiate IFN-β and ISG responses in epithelial and myeloid cells (Karpala et al., 2011; Lee et al., 2020). The concomitant rise in IFN-β and OAS in both EO groups suggests that EOs rapidly prime the PRR–IFN–ISG axis, establishing an antiviral state even in the absence of viral antigen exposure. This interpretation is supported by studies showing that early OAS/ISG induction following TLR stimulation reduces viral replication in avian macrophages, indicating that such transcriptional priming can generate antiviral protection in the absence of detectable pathogen challenge (Santhakumar et al., 2017; Barjesteh et al., 2014). Interestingly, PKR induction occurred only in the *Lippia alba* (T3) group. This pattern aligns with PKR’s dual biology, as it functions both as an IFN-stimulated gene and a direct sensor of dsRNA and stress. Because PKR can be activated independently of broad IFN increases, this mechanistic flexibility provides a plausible explanation for its treatment-specific up-regulation in the absence of broader ISG activation (Dabo and Meurs, 2012; Chaumont et al., 2023).

In the bursa of Fabricius, IFN-β was also up-regulated at day 7 in both EO groups, indicating that early innate antiviral activation occurs not only in mucosal tissues but also in primary lymphoid organs, potentially supporting early antiviral defences and B-cell priming. Indeed, single-cell and tissue studies indicate the bursa harbours IFN-competent stromal and myeloid populations capable of rapid type-I responses, making our observed bursal IFN-β induction biologically plausible and consistent with early lymphoid sensing of immunomodulators or antigens (Shah et al., 2021).

Mechanistically, differences in EO chemotypes likely underlie the observed responses. *Lippia origanoides*, rich in thymol and carvacrol, has been shown to potentiate NF-κB/TLR signaling and early pro-inflammatory activation (Liu et al., 2019; Gholami-Ahangaran et al., 2022), which explains the early TLR3 response in the blend. Conversely, *Lippia alba* (citral/linalool chemotype) appears to moderate inflammation while supporting antiviral readiness, resulting in a more gradual induction of TLR7 and NF-κB. The inclusion of *Rosmarinus officinalis* in the blend likely accentuates immunomodulation through antioxidant diterpenes (carnosic acid, rosmarinic acid), which inhibit cholesterol/ROS-mediated inflammasome activation and reduce IL-1β maturation (Habtemariam, 2023). This combination produces an “antiviral primed but non-excessively inflammatory” state, consistent with the lack of IL-1β up-regulation despite NF-κB activation, reflecting the unique maturation of avian IL-1β (Gyorfy et al., 2003).

Regarding maternal antibody decay, chicks treated with *Lippia alba* essential oil exhibited a significantly slower decline in anti-IBV titers at 7 days post-hatch compared with the blend and control groups. This reduced antibody decay may result from a combination of FcRn-like IgY recycling and enhanced antioxidant or antiviral priming (Okamoto et al., 2024), plausible effects of citral, linalool, and other antioxidant EO metabolites (Brenes and Roura, 2010; Puvača et al., 2022). While direct evidence linking phytochemicals to avian FcRn-mediated IgY recycling is limited, mammalian studies show phytochemical antioxidants can stabilize Ig pools and reduce catabolism, supporting the plausibility of this mechanism in birds and meriting targeted FcRn/IgY studies (Tesar et al., 2008; Abd El-Hack et al., 2021). Given this observation, it is plausible that essential oil bioactives may interact with immune regulatory pathways that influence IgY homeostasis. In this context, avian FcRn homologs protect IgY from lysosomal degradation, thereby extending the antibody half-life. Bioactive EO components could potentially support this pathway, as suggested for other phytochemicals (Tesar et al., 2008; Abd El-Hack et al., 2021). Collectively, these findings suggest that EO chemotypes may influence not only innate immune signaling but also the durability of humoral antibodies during the early post-hatch development period.

From a translational perspective, these findings suggest that EO supplementation can enhance early innate immune readiness even without antigenic stimulation, potentially supporting faster viral control. The stronger responses in the trachea compared to the bursa of Fabricius indicate tissue-specific responsiveness driven by differences in exposure, metabolism, and immune cell composition. Delivery route and formulation are critical modifiers of EO retention; nano-encapsulation or in-ovo application substantially increases tissue exposure relative to unformulated feed or spray, an essential consideration when interpreting tissue-specific transcriptional profiles (Movahedi et al., 2024). The decline in responses by day 14 further underscores the importance of early sampling and optimized delivery strategies. Given the volatility and rapid metabolism of EO components in poultry (Oladokun et al., 2021), limited systemic persistence may explain the more robust effects in mucosal tissues.

Future studies should therefore incorporate earlier sampling intervals, assess EO metabolite pharmacokinetics, and align these with transcriptional and functional immune outcomes to refine EO-based interventions more effectively. Such analyses will help refine EO formulation, chemotype selection, and dosing regimens to exploit their adjuvant-like potential in avian viral control fully.

### EXP2 – NDV vaccinated chickens

In NDV-vaccinated birds, early up-regulation of TLR3 (in both EO groups), TLR7 and NF-κB (in the blend), IL-1β and PKR (in the single), and MDA5 (in both) was observed at day 7 post-vaccination. In contrast, at day 14, only MDA5 remained up-regulated in the single EO group, suggesting a transient but coordinated early innate response. This temporal innate pattern aligned with functional outcomes, as viral load was significantly inhibited in the blend group (*Lippia origanoides, Rosmarinus officinalis*, and *Lippia alba*) group at both day 7 and day 14 dpv. Furthermore, both treatments (blend and single) demonstrated significant anti-NDV antibody production by day 14. Consequently, several controlled studies report that EO or phytochemical supplementation increases HI titers after NDV vaccination and reduces viral shedding, supporting an adjuvant-like effect that complements innate sensor activation (Khalifeh and Abu-Basha, 2014; El-Shall et al., 2020). Taken together, these findings support that EO supplementation can potentiate early innate sensing (via TLR3, TLR7 and MDA5) and downstream antiviral effector activation—particularly in the blend chemotypes—thereby contributing to improved viral control and a more rapid humoral response.

Mechanistically, the up-regulation of TLR3 and MDA5 aligns with their established roles in avian antiviral immunity: TLR3 senses endosomal dsRNA and induces type I IFN, whereas MDA5 serves as the primary cytosolic RNA sensor in chickens (Chen et al., 2013). NF-κB induction in the blend group indicates stronger early pro-inflammatory activation, likely due to TLR-activating terpenes in *Lippia origanoides* and *Rosmarinus officinalis* (Gholami-Ahangaran et al., 2022; Habtemariam, 2023). In contrast, *Lippia alba*, rich in citral and linalool, appears to favor a balanced antiviral–antigen presentation profile (Valdivieso Ugarte et al., 2019). The selective PKR up-regulation in the *Lippia alba* EO group suggests the activation of both interferon-stimulated genes and direct stress/viral RNA sensing pathways (Dabo and Meurs, 2012).

These contrasting immune signatures likely help explain why the superior viral inhibition observed in the blend EO group likely reflects early and broad mucosal innate activation, rather than intracellular effector engagement. In contrast, the single *Lippia alba* treatment exhibited sustained upregulation of MDA5 and PKR, indicating a more prolonged intracellular antiviral signalling profile that did not translate into measurable viral load reduction at the sampled time points (Li et al., 2023). The concurrent elevation of antibody titers in both groups indicates that EOs function as adjuvant-like modulators, enhancing vaccine-induced humoral responses, consistent with previous studies reporting increased NDV antibody titers and reduced viral shedding following EO supplementation (Khalifeh and Abu-Basha, 2014; El-Shall et al., 2020).

Overall, these treatment-specific differences likely reflect chemotype-dependent activation profiles, where the blend induces broad TLR3/TLR7/NF-κB activation, providing early immune stimulation but potentially promoting earlier antigen clearance or heightened inflammation. In contrast, Lippia alba’s more moderate but effective *MDA5/PKR* activation appears to favor intracellular viral control with moderated inflammation (Oladokun et al., 2021).

### EXP3 – IBDV vaccinated chickens

In the bursa of Fabricius, TLR7 up-regulation in the bursa at 7 days post-vaccination, followed by TLR3 and MDA5 activation by day 14, indicates early engagement of antiviral sensing pathways in EO-treated birds. The concurrent reduction in viral load and higher anti-IBDV titers, particularly in the blend group, suggest that EO supplementation enhances innate RNA sensing and accelerates protective humoral responses.

Early TLR7 up-regulation indicates priming of endosomal RNA sensors, likely accelerating type I IFN and interferon-stimulated gene (ISG) induction to establish an antiviral environment conducive to antibody generation (Rauf et al., 2011). Moreover, IBDV protein (VP3) has a high affinity for MDA5, blocking the signalling pathway triggered by IBDV dsRNA. Therefore, since IBDV VP3 binds and antagonizes MDA5, early TLR7 activation may be critical to initiate IFN responses before viral antagonists accumulate; accelerating TLR7 engagement could hence improve early viral control (Ye et al., 2014). Thus, consistent with this early activation, the subsequent up-regulation of TLR3 and MDA5, observed primarily in the blend group, suggests a transition from endosomal to broader cytosolic RNA sensing, reflecting the temporal kinetics and tissue-specific effects of EO stimulation. Consequently, this differential pattern indicates that the enhanced MDA5 response in the blend group may result from a synergistic interaction between rosemary polyphenols and Lippia origanoides terpenes, which are known to potentiate NF-κB and MAVS-mediated signaling (Habtemariam, 2023; Gholami-Ahangaran et al., 2022). Accordingly, the EO blend (*Lippia origanoides, Rosmarinus officinalis*, and *Lippia alba*) appears to promote more effective viral control through coordinated activation of TLR7- and MDA5-mediated pathways, which likely explains the significant viral inhibition observed in association with MDA5 induction.

The significant antibody enhancement in both EO groups aligns with an adjuvant-like modulation of adaptive responses, potentially through improved B-cell activation and plasma cell survival mediated by early innate signaling and antioxidant protection (El-Shall et al., 2020; Sandner et al., 2020; Engida et al., 2023). Differences between treatments reflect distinct chemotypes and bioactive compound profiles, with the blend inducing broader but delayed cytosolic/endosomal PRR activation. At the same time, *Lippia alba* triggers rapid endosomal sensing and earlier viral control. Future studies should include earlier sampling (1–3 days post-vaccination) and quantify EO metabolites in the bursa to correlate with transcriptional and functional immune endpoints.

### EXP4 – IBV vaccinated chickens

In the trachea, OAS was up-regulated in both EO groups at day 7 post-vaccination, followed by increased expression of TLR3 and PKR in both groups at day 14, and MDA5 up-regulation in the single EO group only. Although no significant viral inhibition was detected at either time point, the single EO group exhibited higher anti-IBV antibody titers at day 7.

OAS, an interferon-stimulated gene mediating antiviral RNA degradation via RNase L, is rapidly induced following type I IFN signaling or PRR activation (He et al., 2024). Thus, its early induction suggests that EO supplementation may prime antiviral effectors even before robust activation of upstream sensors. The subsequent up-regulation of TLR3 and PKR likely reflects a progression from effector priming to sensor-driven antiviral signaling (Kameka et al., 2014; Dabo and Meurs, 2012). However, the absence of viral load reduction could indicate that the timing or magnitude of upstream activation was insufficient for measurable inhibition. Finally, the higher antibody titers in the blend group at day 14 likely result from enhanced TLR/NF-κB signaling and antioxidant synergy contributed by thymol, carvacrol, carnosic, and rosmarinic acids (Gholami-Ahangaran et al., 2022; Habtemariam, 2023), whereas *Lippia alba* alone appears to favor early antiviral effector activation with a delayed humoral response.

These results emphasize the importance of assessing both early antiviral effector induction and upstream sensor activation when evaluating EO immunomodulation. Reviews on EOs in poultry highlight that bioavailability, formulation (e.g., nanoencapsulation), and tissue retention are significant determinants of immunomodulatory efficacy (Movahedi et al., 2024). Future studies should include very early time points (1–3 days), kinetics of sensor activation (TLR3, MDA5, TLR7) alongside effector genes (OAS, PKR), quantification of EO component concentrations in mucosal tissues, and evaluation of viral replication kinetics beyond day 14 to capture the EO-virus-host interaction fully.

### Comparative summary of EO effects across experiments 1–4

Across experiments, several consistent patterns emerged. **Tissue specificity** was evident, as the trachea consistently showed stronger and earlier antiviral responses than the bursa (EXP1 and EXP4), likely due to higher EO exposure and richer PRR expression. In contrast, bursal activation was more virus-dependent, particularly during IBDV vaccination (EXP3). **Chemotype-dependent immune profiles** were also evident: the EO blend induced broad and early activation of TLR3, TLR7, and NF-κB, resulting in stronger viral inhibition in NDV and IBDV models. In contrast, *Lippia alba* triggered more targeted intracellular antiviral signaling—especially MDA5 and PKR—leading to measurable improvements in early antibody responses but less consistent viral suppression. **Virus-specific patterns** further differentiated outcomes, with NDV and IBDV showing clear improvements in viral control following blend treatment. In contrast, in IBV-vaccinated birds, EO-mediated antiviral priming occurred but did not reach the magnitude needed for measurable viral reduction. Finally, **temporal dynamics** were consistent across studies, with most EO-driven immune effects peaking at day 7 and waning by day 14, underscoring the importance of early sampling and refined delivery strategies. Collectively, these findings demonstrate that essential oils function as **selective immunomodulators**, priming innate antiviral pathways and enhancing vaccine-induced humoral responses in a manner that is dependent on chemotype, tissue, virus, and timing.

## Conclusion and future directions

Overall, EO supplementation modulated innate antiviral signaling, viral control, and adaptive humoral responses in a chemotype- and tissue-specific manner. *Lippia alba* promoted rapid endosomal sensing and early viral inhibition, whereas the blend induced broader NF-κB/TLR activation, supporting sustained antiviral readiness and antibody enhancement. EO volatility, tissue retention, and chemotype composition influenced both the magnitude and timing of responses. Future studies should focus on early post-treatment sampling (1–3 days), quantification of EO metabolites in relevant tissues, formulation strategies to enhance bioavailability (e.g., nanoencapsulation), and combinatorial EO–vaccine experiments to optimize immune outcomes. These approaches will refine the use of EOs as immunomodulatory agents in poultry production.

## Author contributions

Conceptualization, J.C.R.-L., J.J., and J.A.-I.; investigation, J.A.-I., M.F.N.-O., A.L.M., B.C.M., and D.S.V.-B.; writing - original draft preparation, J.A.-I., J.J. and J.C.R.-L.; supervision, J.C.R.-L., J.J., and B.C.M.; project administration, A.L.M., and B.C.M. All authors have read and agreed to the published version of the manuscript.

## Funding

This research was funded by Promitec de Colombia, by the Facultad de Medicina Veterinaria y de Zootecnia, Universidad Nacional de Colombia, Sede Bogotá (funding number Hermes 56489 - 2022) and by Atlantic Veterinary College, University of Prince Edward Island, Charlottetown, Canada (Grant NSERC-Discovery A610129).

## CRediT authorship contribution statement

**Jaime A. Angel-Isaza:** Writing – original draft, Investigation, Conceptualization. **María F. Naranjo-Ortiz:** Investigation. **Angi L. Montoya:** Project administration, Investigation. **Diana S. Vargas-Bermúdez:** Investigation. **Blanca C. Martínez:** Supervision, Project administration, Investigation, Funding acquisition. **Jairo Jaime:** Writing – original draft, Supervision, Funding acquisition, Conceptualization. **Juan C. Rodríguez‑Lecompte:** Writing – review & editing, Supervision, Funding acquisition, Conceptualization.

## Disclosures

Jaime Angel-Isaza, Angi L. Montoya and Blanca Cecilia Martinez are employed by the company Promitec. The remaining authors declare that the research was conducted in the absence of any commercial or financial relationships that could be construed as a potential conflict of interest.
